# Developing a curriculum for health professional students on point of care testing for medical diagnosis

**DOI:** 10.5116/ijme.5780.a9cd

**Published:** 2016-08-13

**Authors:** Rooyen Tinago Mavenyengwa, Terence Nyamayaro

**Affiliations:** 1 Department of Medical Microbiology, University of Zimbabwe, College of Health Sciences, Harare, Zimbabwe; 2 Department of Chemical Pathology, University of Zimbabwe College of Health Sciences, Harare, Zimbabw

**Keywords:** Curriculum, health professional students, point of care testing, medical diagnosis

## Introduction

The University of Zimbabwe College of Health Sciences runs a Bachelor of Medicine and Surgery (MBChB) five-year degree programme in the training of medical doctors. The MBChB curriculum has some gaps in the teaching of point of care tests (POCTs) despite their increased use in the country. The point of care testing refers to medical diagnostic testing done by operators with no formal training in laboratory sciences at the site of patient care.^1^

The point of care tests give quick results and can be performed outside of the conventional diagnostic laboratory. Besides being user-friendly and affordable, the instruments are easily transportable. Pre-analytic and post-analytic errors are often low, and small sample volumes are used.^2^

Despite the advantages and potential of POCTs, the current medical training in Zimbabwe does not adequately equip health professional students on all aspects of POCTs. Provision of new diagnostic tests should be complemented with training students on the available commercial POC tests. The huge health demands due to the increasing population and the effects of HIV and AIDS in the country, have increased the need to decentralize healthcare services outside traditional settings.^3^ Proper training of medical students in POCTs leads to high-quality patient care. Full exploitation of this potential is often hampered by unsatisfactory test performance, quality control and regulatory issues, and inadequate training of personnel.^4^ Educators must manage the challenge of training through curriculum review. This would address the lack of knowledge, attitudes and skills in POCT testing which often limits the extensive and effective use of such tests.

Some health care providers in Zimbabwe lack the necessary training in POCT technology due to little attention being devoted to curriculum adjustment to accommodate POCTs. The initiative to use the tests has been driven by manufacturers and non-governmental organizations instead of medical diagnostic laboratories. The purpose of this perspective is to describe the development and initial implementation of a curriculum for teaching medical students at the point of care tests for diagnosis of medical conditions at the University of Zimbabwe College of Health Sciences for the MBChB programme.

### Needs assessment

The six-step approach for developing curricula for medical education designed by Kern and colleagues,was followed.^4^ Review of the published literature and perusal of the current medical curriculum at the University was done to assess the existence of components in the teaching of POCTs in medical diagnosis. The gaps in the teaching of POCTs in the current University of Zimbabwe College of Health Sciences curriculum were identified. Lecturers within the College’s pathology and clinical departments and stakeholder organizations were consulted to gather their views on various aspects of POCT. The organizations included nurses, clinicians, medical laboratory scientists, including the Medical Laboratory and Clinical Scientists Council (MLCSC), health personnel from the Harare City Health Department, health diagnostic laboratories and personnel from the Ministry of Health and Child Care (MHCC) Diagnostic Service Department. The needs assessment focused on POCT storage facilities, safety, acceptance, quality assurance and control, personnel competency, main test failures, evaluation of testing kits and devices, registration with the Health Professions Council and POCTs knowledge.  The assessment involving the lecturers focused on identifying the learning objectives, content, activities, study resources and diagnostic, formative and summative assessment strategies. The survey was approved by the Joint Parirenyatwa Hospital and College of Health Sciences Joint Ethics Committee.

### Curriculum

The main goal of the curriculum was identified as ensuring that students acquire the requisite knowledge, attitudes, skills and confidence to utilize POCTs in their practice. The learning objectives included ensuring that students have the ability to identify  and describe different types of POCTs appropriate for Zimbabwe‘s health services, select the most suitable POCTs taking into account their predictive values, diagnostic specificities and sensitivities, identify where,  when and how to use the tests, explain the  scientific principles behind POCTs, describe procedures for their implementation, and the essential quality assurance and quality control requirements, correctly perform and interpret tests, observe ethics and be able to maintain stock for POCTs consumables. The teaching strategies identified were the lecture method, small group discussions, and e-learning. Videos could allow students to visualize POCT protocols and understand how to troubleshoot errors. Following standard operating procedures and direct observation of instructor demonstration of medical procedures could also be used.

Laboratory practicals sessions allow students to practice procedures and learn how to interpret the tests. This promotes self-directed study, stimulates the development of psychomotor skills and improves retention. Small project assignments could equip students with the ability to select an appropriate POCT, develop an SOP, validate and use the test for the relevant case scenario and compile reports. Compiling log books summarizes the techniques they would have mastered.

The assessment strategies included the use of multiple choice questions, short structured questions, practical examinations and competency tests. Assessment of psychomotor skills through direct observation of test performance could be done if instructors are available.  Final competency assessment using blinded specimens could also be useful.

## Conclusions

This POCT curriculum should be introduced immediately following consultations with preclinical, pathology and clinical departments to compile the specific content, teaching framework and comprehensive teaching activities and resources. Point of care tests are necessary diagnostic tools for use in Zimbabwe’s health system whose aspects should be a distinct component of the MBChB programme.

### Acknowledgements

We acknowledge the assistance received from the Novel Education Clinical Trainees and Researchers (NECTAR) Programme and the Medical Education Partnership Initiative (MEPI) who funded the training of the Health Education Advanced Leadership Programme.

### Conflicts of Interest

The authors declare that they have no conflict of interest.

## References

[1] Nichols JH (2007). Point of care testing.. Clin Lab Med.

[2] Louie RF, Tang Z, Shelby DG, Kost GJ (2000). Point-of-Care Testing: Millennium Technology for Critical Care.. Lab Med.

[3] Schito M, Peter TF, Cavanaugh S, Piatek AS, Young GJ, Alexander H, Coggin W, Domingo GJ, Ellenberger D, Ermantraut E, Jani IV, Katamba A, Palamountain KM, Essajee S, Dowdy DW (2012). Opportunities and challenges for cost-efficient implementation of new point-of-care diagnostics for HIV and tuberculosis.. J Infect Dis.

[4] Nichols JH, Christenson RH, Clarke W, Gronowski A, Hammett-Stabler CA, Jacobs E, Kazmierczak S, Lewandrowski K, Price C, Sacks DB, Sautter RL, Shipp G, Sokoll L, Watson ID, Winter W, Zucker ML (2007). Executive summary. The National Academy of Clinical Biochemistry Laboratory Medicine Practice Guideline: evidence-based practice for point-of-care testing.. Clin Chim Acta.

[5] Kern DE, Thomas PA, Howard DM, Bass EB. Curriculum development for medical education: a six-step approach. Baltimore: Johns Hopkins University Press; 2009.

